# Normative Application of Xiyanping Injection: A Systematic Review of Adverse Case Reports

**DOI:** 10.1155/2018/4013912

**Published:** 2018-11-15

**Authors:** Shiqi Chen, Joey S. W. Kwong, Rui Zheng, Yanping Wang, Hongcai Shang

**Affiliations:** ^1^Key Laboratory of Chinese Medicine of Ministry of Education and Beijing, Dongzhimen Hospital Affiliated to Beijing University of Chinese Medicine, Beijing 100700, China; ^2^Jockey Club School of Public Health and Primary Care, Faculty of Medicine, The Chinese University of Hong Kong, Hong Kong; ^3^Institute of Basic Research in Clinical Medicine, China Academy of Chinese Medical Sciences, Beijing 100700, China

## Abstract

**Purpose:**

To summarize the characteristics and the relevant factors and to give references for preventing adverse drug reactions (ADRs) associated with xiyanping (XYP), we provide a systematic review of adverse case reports about XYP.

**Methods:**

Seven medical databases were searched from inception to January 2018. Case reports detailing ADRs associated with XYP were included. Data were extracted independently by two reviewers. After the assessment of causality and severity, we carried out a descriptive analysis for the relevant ADRs.

**Results:**

Forty-three articles involving a total number of 55 cases were included. Eight cases were off-label drug use. In the remaining 47 cases, 26 (55.3%) had probable causality and 23 (48.9%) were serious cases. XYP used in children (≤14 years old) accounted for 66.0%. Respiratory diseases (83.0%) were major primary diseases. No allergic history mentioned (55.3%) and unspecific drug combination (59.6%) were common in these reports. As for ADR types, anaphylaxis and anaphylactic shock were up to 97.9%. ADRs happened mostly when applying XYP within 30 minutes (70.2%) and the majority (95.7%) were cured when treated in time.

**Conclusions:**

Clinicians and patients are supposed to obey the package insert of XYP in clinical application. Through the results of XYP, normalization of ADR reports is also worthy of attention. High-quality researches are required to improve the drug instruction and evaluate the safety of XYP in effective diseases and different age groups. Mechanism of ADRs aiming at the hypersensitivity and the drug combination should still be further identified.

## 1. Introduction

Xiyanping (XYP), a traditional Chinese medicine available in injection form, is used for the treatment of bronchitis, hand, foot, and mouth disease, bacillary dysentery, and other infectious diseases in China [1–4]. The sole active ingredient of XYP is the water-soluble andrographolide [5], which is a diterpenoid lactone from the traditional Chinese medicinal herb* Andrographis paniculata* [3]. Data have showed the effects of this sulfonated andrographolide in sepsis, acute lung injury, the associated antimicrobial, antiviral, and other anti-inflammatory actions in representative studies [5–8]. In China, XYP has been a topic [9, 10] since the safety impacted factors are not completely clear and the mechanism of ADRs is not validated. Therefore, it is imperative to conduct comprehensive assessment of ADRs associated with XYP using rigorous systematic review of case reports [11] to fully understand the scope of safety of XYP and the prevention measures in clinical use.

## 2. Methods

### 2.1. Study Identification

We searched PubMed, Embase, Cochrane Library, China National Knowledge Infrastructure (CNKI), Chinese Scientific Journal Database (VIP), Chinese Biomedical Literature Database (CBM), and Wanfang Data from database inception to 9 January 2018 for eligible case reports with information regarding ADRs related to XYP in humans published peer-reviewed journals. No restrictions on language were imposed. The search terms, in both English and Simplified Chinese, included “andrographolide sulfonate”, “xiyanping”, “adverse drug reaction”, “adverse effect”, “side-effect”, “anaphylaxis”, “allergic reaction”, “safety”, “toxicity”.

### 2.2. Study Selection and Data Extraction

Two reviewers (Shiqi Chen and Rui Zheng) independently conducted the study selection and data extraction. Firstly, we removed the duplicates of all papers through searching. Then, we examined the titles and abstracts according to the inclusion criteria. Finally, the full texts were retrieved for detailed assessment. A standardized data extraction form was used to collect data of included studies, including title, first author, year of publication, drug batch number, admission time, age, gender, primary disease, allergic history, solvent, dosage, drug combination and mixture, dripping speed, ADR type, ADR symptom, occurrence time, ADR treatment, and prognosis. Disagreements were resolved through discussion or by consulting a third reviewer (Hongcai Shang).

### 2.3. Criteria of ADR, Causality, and Severity

Two investigators (Shiqi Chen and Rui Zheng) evaluated ADRs of included studies according to the criteria of ADR, causality, and severity. The definition of ADR by China Food and Drug Administration is “an unintended and unfavorable outcome that occurs during or after the normal use of a qualified pharmaceutical” [12]. Further investigation was required to clarify any casual relationship between the case report findings and XYP. The reports that were not coincident with the definition of ADR or irrelevant with XYP were not analyzed.

The causality categories described by the WHO Collaborating Centre for International Drug Monitoring (The Uppsala Monitoring Centre) [13, 14] are classified into 6 levels: certain, probable, possible, unlikely, conditional/unclassified, and unassessable/unclassifiable ADRs related to the drug. The results consist of 5 aspects: (1) the clinical events with laboratory test abnormality; (2) the plausible time between drug use and ADR; (3) the plausible explanations provided by other drugs, chemicals, or underlying diseases; (4) the response to withdrawal of the drug (dechallenge); and (5) the rechallenge information. The detailed classification standard is showed in Table 1 [14].

Serious ADRs can be identified as one of the following 6 reasons [12]: (1) lethality; (2) life-threatening; (3) carcinogenesis, teratogenesis, or birth defects; (4) conspicuous or permanent injuries or organ dysfunctions; (5) prolonged length of hospitalization; and (6) other serious medical incidents which can progress to (1)-(5) consequences above.

### 2.4. Data Analysis

Descriptive analysis was applied to the included studies involving 5 aspects: (1) age and gender; (2) primary diseases and allergic history; (3) mixture and combination of drugs; (4) occurrence time and type of ADR; and (5) prognosis of ADR.

## 3. Results

### 3.1. Studies Identified and Characteristics

**Figure 1:**
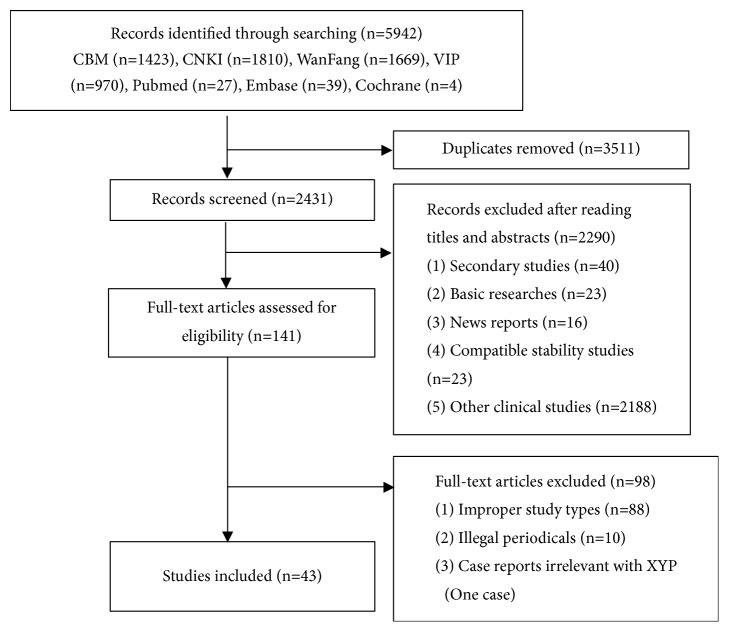
Process of searching and screening studies.

The initial database search yielded 5942 records. After removing 3511 duplicates, 2431 records were screened for eligibility. Of these, full-text articles of 141 studies were obtained for further assessment. We eventually included 43 reports (involving 55 cases). Our study selection process is illustrated in Figure 1. The characteristics of the case reports are shown in Table 2.

### 3.2. Assessment Results of ADR, Causality, and Severity

Administration of XYP in 8 cases was found to not be in line with the package inserts of XYP: 4 were outside of the recommended dripping speed and 4 used improper solvents; thus they were excluded from our ADR analysis. After the assessment of causality and severity for the remaining 47 cases (reported in 36 articles), the results of causality showed that 26 (26/47, 55.3%) were probable and 21 (21/47, 44.7%) were possible. For the severity, it showed that 23 (23/47, 48.9%) were serious ADRs while 24 (24/47, 51.1%) were general ADRs.

#### 3.2.1. Primary Diseases and Allergic History

Respiratory diseases (83.0%, 39/47) were major primary diseases, including upper respiratory tract infections, acute/chronic bronchitis, bronchopneumonia, amygdalitis, pneumonia, bronchial asthma, bronchiectasia with infections, and mycoplasma pneumonia. 4.3% (2/47) were fever of unknown origin. The rest are digestive and skin diseases, comprising about 12.8% (6/47). The details were given in Table 3.

In 47 cases, 26 were unspecific in allergic history, making up 55.3% (26/47). The patients of nonallergic history were 14 (14/47, 29.8%). The other 7 cases (7/47, 14.9%) had the allergic history. Detailed data were shown in Table 4.

#### 3.2.2. Mixture and Combination of Drugs

All included ADR cases of XYP failed to report the mixture of drugs. There were 28 (28/47, 59.6%) cases which were unclear of the combination. In terms of the combination use, 3 drugs were 5 (5/47, 10.6%) and 2 drugs were 9 (9/47, 19.1%). The remaining 5 cases (5/47, 10.6%) were single use with XYP injection. Details of other combined drugs were shown in Table 5.

#### 3.2.3. Occurrence Time and Types of ADR

Amongst the included case reports, ADRs of 17 cases (17/47, 36.2%) occurred in 5 minutes, and 16 (16/47, 34.0%) were between 5 minutes and half an hour. Two cases (2/47, 4.3%) were between half an hour and 3 hours, and the remaining 12 cases (12/47, 25.5%) did not report the occurrence time.

For the ADR type, anaphylaxis and anaphylactic shock were common types, up to 97.9%. 29 (29/47, 61.7%) cases were treated as anaphylaxis and 17 (17/47, 36.2%) were anaphylactic shock. Patients of anaphylaxis may break out in a rash, together with itch, or they may have symptoms such as cyanosis, cough, abdominal pain, dizziness, chest congestion, short of breath, chills, or fever. Anaphylactic shock was more serious than anaphylaxis. Besides the symptoms above, it had a sharp drop of blood pressure, dyspnea, and disturbance of consciousness. In addition to these cases, 1 (1/47, 2.1%) was just reported to have dizziness of unknown origin. More details were in Table 6.

#### 3.2.4. Prognosis of ADR

Oxygen uptake, epinephrine, dopamine, dexamethasone, diphenhydramine, promethazine, and 10% calcium gluconate were frequently used in anaphylaxis and anaphylactic shock as described in the case reports. After the treatment, 45 cases (45/47, 95.7%) recovered soon.

The case of ADR resulting in death was having an allergy and had primary disease of scald with infections [33]. After the first-time treatment, the right upper limb was swelling and painful to exercise. The next day after the second-time use of XYP, the patient developed into anaphylactic shock, showing dyspnea, disturbance of consciousness, and a sharp drop of blood pressure. After the urgent anti-shock treatment, the patient died finally. The report had recorded that sulfonamide was his allergen and at the same time had a 10-year history of diabetes mellitus. However, it was unclear of the allergic constitution and the disease progression. With the rational relationship of time between drug use and ADR, the causality can be classified as possible. The other case of anaphylactic shock leading to the vegetative state had the primary disease of pediatric asthmatic bronchitis [24]. The symptoms of shock came out at the first time when using XYP. After the anti-shock treatment, the patient was in a coma and finally in the persistent vegetative state. Allergic history was indistinct. Considering rational relationship of time, absence of both the provocation test (rechallenge), and the clinically reasonable response on withdrawal (dechallenge), the causality can be also classified as possible. The cases which are classified possible need further investigating in the concurrent diseases or other drugs or chemicals. Other factors like these may be more possible than XYP itself.

## 4. Discussion

### 4.1. Normalization of the Drug Use and the ADR Reports

According to the results of a prospective, postmarketing, and large-scale centralized hospital monitoring study [58], a total of 30759 patients employing XYP were collected from 21 hospitals; as a result, a number of 23 patients developed mild ADRs related to XYP, and the ADR incidence rate was 0.75‰ (95% confidence interval: 0.47‰ ~ 1.12‰). Another prospective randomized controlled trial [2], which contained 114 patients of severe hand, foot, and mouth disease in XYP combination group, observed no ADRs during the period of study. Considering the rare ADR incidence of XYP, we should pay more attention to the rational drug use in clinical practice.

Our comprehensive literature search revealed 8 cases where the use of XYP fell outside of the recommendations as provided in the package inserts of XYP, including fast dripping speed and the use of improper solvents. Formalization of the drug use should be emphasized for fear of an increased risk of adverse events. Overdose and overspeed would quicken blood circulation and increase cardiac burden, leading to heart failure and serious anaphylactic shock [59]. Clinicians and patients should strictly follow the instruction to assure the safety of XYP [60].

We intended to identify detailed information about ADRs through the findings in case reports. However, they were far from content without explicit description and critical appraisal of evidence. Compared with adverse effects and adverse events, information regarding ADRs should be more specific to a drug [61]. In the published literature, assessment of ADR, causality, and severity should be evaluated aforehand and described normatively according to the standard [12–14].

### 4.2. Attention to the Patients Employing XYP

XYP is widely used in various age groups, especially in pediatrics [62]. Kids (≤14 years old) accounted for 66.0% among cases of ADRs. Clinicians and patients should attach importance to the children in early growth with incomplete functional organs, which have individual limits and narrow efficacy of threshold [63].

XYP is an injectable traditional Chinese medicine, with mechanisms of actions in clearing heat and detoxicating for bronchitis, hand, foot, and mouth disease, bacillary dysentery, and other infectious diseases. In these reported cases, it applied not only to some other diseases such as bronchial asthma, adenomesenteritis, and psoriasis, but also to emesis, hypogastralgia, and fever which were not diagnosed definitively. The application of TCMI should be distinguished from the western medicine: treatment based on syndrome differentiation should integrate with the treatment of disease differentiation [64]. According to TCM theory and high-quality research findings, clinical indications of employing XYP should be identified clearly in the package insert.

The unknown information of allergic history in ADR cases accounted for 55.3%. The types of ADRs were mainly allergic reactions, involving diseases in respiratory system, integumentary system, digestive system, and so on. With the high rates of anaphylaxis and anaphylactic shock (97.9%), it is significant to inquire the detailed allergic history from the patients and consider the allergic constitution before taking XYP [65]. Moreover, we need to pay more attention to the allergic histories of andrographis preparations such as lianbizhi injection [66], yanhuning injection [67], and chuanhuning injection [68].

### 4.3. The Mechanism and Prognosis of Allergic Reaction

Allergic reactions without specific proof of diagnosis accounted for 97.9% in these cases. These ADRs happened mostly in 30 minutes (70.2%) and can be defined as immediate hypersensitivity reactions (IHRs) [69]. Previous research has identified that only a small part of those IHRs are immune-mediated (IgE or T cell) IHRs, thus true drug allergies, and the majority are non-immune-mediated IHRs, hence pseudo drug allergies [70]. Studies regarding XYP demonstrated that the results of active systemic anaphylaxis (ASA) test and passive cutaneous anaphylaxis (PCA) test were both negative on guinea pigs [71], while rats receiving directly intracutaneous injection showed positive outcomes with the assay of Evans blue spots [72]. IHRs induced by XYP usually occurred without the previous exposure, conforming to the features of pseudo-allergic reactions [73]. Another TCMI named Shuanghuanglian (SHL) has indicated that during sensitization the specific IgE was not elicited and the pseudo-allergic reactions were directly motivated by the activation of RhoA/ROCK signal pathway [74]. In-depth mechanism of the hypersensitivity reaction for XYP merits future researches to seek potential therapeutic strategy to prevent or treat with the associated ADRs.

After the expectant treatment for ADRs, 95.7% cases were improved and recovered. It has been warned in the header of the package insert that people should employ XYP in hospitals with emergency equipment in case of the anaphylactic shock.

### 4.4. Tube-Flushing and Systematic Researches in Combination of Drugs

The package insert of XYP has showed that mixture of drugs should be forbidden [75], and combination of drugs should be adopted prudently because the possible insoluble particles precipitated out would produce ADRs [76]. The drug instruction has also informed that appropriate intervals associated with tube-flushing should be vigilant in case of drug interactions. However, a study [77] concerning 3 TCMIs (including XYP, reduning injection, and danhong injection) showed that only 9.6% of the 2,045 investigating cases were flushed or replaced with infusion tubes at the time of intervals. Due to XYP-use in infectious diseases [1–3], most of the combined medications were antibiotics and antiviral drugs in clinical practice. In this study, more than a half of the cases did not even report the combinations. Researchers should record detailed information about combined medication in the case reports and strengthen the systematic researches in the incompatibility of antibiotics and antiviral drugs.

### 4.5. Limitation

The systematic review of case reports has some limitations itself. In a systematic review of RCTs, we can conduct a meta-analysis to identify the efficacy of a drug or an intervention. However, in a systematic review of case reports, we cannot use the combined data to obtain advantages or disadvantages for the missing number of drug users and comparisons. So, we just carried out a descriptive analysis and list all the factors for the relevant ADRs in different states. Afterwards, we expect more prospective clinical studies and experiment researches to obtain the accurate ADR outcomes in every state.

## 5. Conclusions

As for the rare incidence of ADRs of XYP in the prospective study, clinicians and patients should strictly obey the drug instruction in clinical practice, including indications, allergic history, solvent, dosage, drug combination, dripping speed, and tube-flushing.

ADRs in the case reports merit close attention and detailed description. Assessment of ADR, causality, and severity are all necessary in reporting an ADR case, and more valid information from the literatures are also required to construct the integral postmarketing security evaluation system [78].

Considering its misleading indications and unclear age groups in the package insert, high-quality clinical studies and pharmaceutical experiments are demanded to supplement the drug instruction. Attention should be paid when XYP is used in children and people with allergic constitution. To prevent and treat with serious anaphylactic shock, mechanism of the hypersensitivity reaction and the drug combination should still be fully identified.

## Figures and Tables

**Table 1:** The causality categories described by the WHO Collaboration Center for International Drug Monitoring (The Uppsala Monitoring Centre) [14].

**Degrees**	**Definitions**
Certain	A clinical event, including laboratory test abnormality, occurring in a plausible time relationship to drug administration, and which cannot be explained by concurrent disease or other drugs or chemicals. The response to withdrawal of the drug (dechallenge) should be clinically plausible. The event must be definitive pharmacologically or phenomenologically, using a satisfactory rechallenge procedure if necessary.

Probable/Likely	A clinical event, including laboratory test abnormality, with a reasonable time sequence to administration of the drug, unlikely to be attributed to concurrent disease or other drugs or chemicals, and which follows a clinically reasonable response on withdrawal (dechallenge). Rechallenge information is not required to fulfil this definition.

Possible	A clinical event, including laboratory test abnormality, with a reasonable time sequence to administrations of the drug, but which could also be explained by concurrent disease or other drugs or chemicals. Information on drug withdrawal may be lacking or unclear.

Unlikely	A clinical event, including laboratory test abnormality, with a temporal relationship to drug administration which makes a causal relationship improbable, and in which other drugs, chemicals or underlying disease provide plausible explanations.

Conditional/Unclassified	A clinical event, including laboratory test abnormality, reported as an adverse reaction, about which more data is essential for a proper assessment, or the additional data is under examination.

Unassessable/Unclassifiable	A report suggesting an adverse reaction which cannot be judged because information is insufficient or contradictory, and which cannot be supplemented or verified.

**Table 2:** Patient details recorded from the included studies.

**Case report**	**Drug batch number**	**Occurrence time (year/month/ day)**	**Age/Gender (years/female or male)**	**Primary disease**	**Dosage (mL)**	**Dripping speed (**drops per minute**)**	**Drug Delivery**	**Solvent**	**Combination**	**Mixture**	**Allergic history**	**Medication interval**	**ADR type**
Danzhu Huang et al. [15]	NA	2008.6.19	0.75/M	Diarrhea	NA	NA	i.v.gtt.	NA	NA	No	NA	NA	Anaphylactic shock
Wenlan Huang et al. [16]	NA	2015.2.1	65/F	Upper respiratory tract infections	10	25	i.v.gtt.	②	NA	No	No	2min	Anaphylactic shock
Bo Chen et al. [17]	20070606	NA	28/F	Acute bacillary dysentery	12	NA	i.v.gtt.	①	No	No	Cephalosporin	8min	Anaphylactic shock
Xiangyu Di et al. [18]	NA	NA	7/M	Mycoplasma pneumonia	4	NA	i.v.gtt.	③	NA	No	NA	NA	Anaphylaxis
Hong Lai et al. [19]	NA	2012.7	6/M	Upper respiratory tract infections	NA	NA	i.v.gtt.	NA	Ceftezole	No	No	NA	Anaphylaxis
Zhongying Jian et al. [20]	NA	NA	80/M	Chronic bronchitis	8	50	i.v.gtt.	②	NA	No	No	2min	Anaphylaxis
Zhong Miao et al. [21]	2014031103	NA	1/M	Bronchitis	2	NA	i.v.gtt.	①	Mezlocillin sodium	No	No	After the infusion	Anaphylactic shock
Shujun Hu et al. [22]	NA	NA	8/M	Upper respiratory tract infections	4	40	i.v.gtt.	①	NA	No	NA	5min	Anaphylactic shock
Yingyun Shen et al. [23]	20081203	NA	8/M	Upper respiratory tract infections	4	NA	i.v.gtt.	①	NA	No	NA	50mL remained	Anaphylaxis
Yanli Li et al. [24]	20120904	2012.10.15	2/M	Asthmatic bronchitis	3	NA	i.v.gtt.	NA	No	No	NA	20min	Anaphylactic shock (vegetative state)
Hongmei Li et al. [25]	NA	2012.11.23	9/M	Upper respiratory tract infections	3	NA	i.v.gtt.	①	NA	No	NA	50mL remained	Anaphylactic shock
Xiangping Li et al. [26]	20050708	2005.12.18	6/M	Upper respiratory tract infections	2	NA	i.m.	No	No	No	NA	5min	Anaphylaxis
Wenhua Zhang et al. [27]	NA	NA	15/F	Upper respiratory tract infections	6	NA	i.v.gtt.	①	NA	No	NA	1/4 infused	Anaphylaxis
Yuzhi Zuo et al. [28]	051133708	2007.10.12	10/M	Fever of unknown origin	6	NA	i.v.gtt.	①	Penicillin, energy mixture	No	NA	30min	Anaphylaxis
Xinhua An et al. [29]	2013122303	2014.2.7	3/F	Acute bronchitis	2	30	i.v.gtt.	①	Cefuroxime sodium, ribavirin injection	No	No	5min	Anaphylactic shock
Jihong Sun et al. [30]	NA	NA	11/M	Amygdalitis	4	NA	i.v.gtt.	NA	Cefotaxime, Xingnaojing injection	No	No	Time of using Xingnaojing injection (after XYP)	Anaphylaxis
Yuxin Liu et al. [31]	NA	2014.3.20	53/F	Amygdalitis	10	60	i.v.gtt.	①	Azithromycin	No	No	NA	Convulsion of unknown origion
Kunna Xiu et al. [32]	NA	NA	NA/F	Upper respiratory tract infections	5	NA	i.v.gtt.	①	NA	No	NA	2min	Anaphylaxis
Hong Yu et al. [33]	NA	2002.12.31	60/M	Scald with infections	12	NA	i.v.gtt.	NA	NA	No	Sulfonamides	3h	Anaphylactic shock (death)
Xiaolei Chen et al. [34]	20090914	2010.1.17	34/F	Upper respiratory tract infections	4	NA	i.v.gtt.	①	No	No	No	5min	Anaphylactic shock
Xiwei Feng et al. [35]	NA	NA	2/F	Bronchopneumonia	2	NA	i.v.gtt.	①	NA	No	NA	15min	Anaphylaxis
Hualing Qiao et al. [36]	NA	NA	16/F	Upper respiratory tract infections	8	NA	i.v.gtt.	①	NA	No	NA	10min	Anaphylactic shock
Taiyu Wan et al. [37]	NA	2001.7.3	30/F	Upper respiratory tract infections	10	40	i.v.gtt.	④	Dexamethasone, VC	No	NA	2min	Anaphylactic shock
Xiaojie Hao et al. [38]	NA	NA	5/F	Upper respiratory tract infections	3	NA	i.v.gtt.	③	NA	No	No	5min	Anaphylactic shock
Xiaoru He et al. [39]	20100603	NA	10/F	Mycoplasma pneumonia	6	NA	i.v.gtt.	①	Azithromycin, cefoperazone	No	NA	20min	Anaphylaxis
Cuiling Wang et al. [40]	NA	2005.6.5	3.5/M	Adenomesenteritis	2	NA	i.v.gtt.	NA	No	No	Cephalosporin	5min	Anaphylaxis
Liqiang Wu et al. [41]	2013020602	2013.9.19	39/M	Upper respiratory tract infections	12	NA	i.v.gtt.	①	NA	No	No	5min	Anaphylactic shock
Xiaoqiang Xiong et al. [42]	20111024	2012.2.16	3/M	Amygdalitis	2	NA	i.v.gtt.	①	NA	No	NA	15min	Anaphylactic shock
Fengshu Zhang et al. [43]	20090911	2009.2.6	1/F	Upper respiratory tract infections	2	45	i.v.gtt.	①	NA	No	No	8min	Anaphylactic shock
Guixin Zhang et al. [44]	NA	2008.2.18	3.5/F	Amygdalitis	4	NA	i.v.gtt.	①	NA	No	No	5min	Anaphylaxis
Yunying Zhang et al. [45]	20090715	NA	51/F	Pneumonia	8	NA	i.v.gtt.	①	NA	No	No	20min	Anaphylactic shock
Fang Dong et al. [46]	NA	NA	12/M	Amygdalitis	8	NA	i.v.gtt.	①	NA	No	NA	10min	Anaphylactic shock
Fang Dong et al. [47]	060417	NA	0.83/M	Bronchitis	2	NA	i.v.gtt.	①	NA	No	NA	5min	Anaphylaxis
Xuejin Zhao et al. [48]	20040918	2004.10.18	58/F	Chronic bronchitis	8	50	i.v.gtt.	②	No	No	No	5min	Anaphylaxis
Li Zheng et al. [49]	20131015	NA	55/F	Pneumonia	8	NA	i.v.gtt.	①	NA	No	No	20min	Anaphylactic shock
Li Zheng et al. [49]	20140105	NA	4/M	Bronchitis	4	NA	i.v.gtt.	①	NA	No	Yanhuning injection	10min	Anaphylactic shock
Liwei Yang et al. [50]	NA	2012.3.29	4/F	Bronchitis	2	NA	i.v.gtt.	①	NA	No	NA	NA	Anaphylaxis
Liwei Yang et al. [50]	NA	2012.8.6	7/M	Pneumonia	4	NA	i.v.gtt.	①	NA	No	NA	20min	Anaphylaxis
Xiuju Wang et al. [51]	NA	2014.6.9	64/F	Pneumonia	20	NA	i.v.gtt.	①	NA	No	Penicillins	15min	Anaphylaxis
Xiuju Wang et al. [51]	NA	2014.6.23	35/F	Bronchiectasia with infections	20	NA	i.v.gtt.	①	NA	No	Cephalosporin	20min	Anaphylaxis
Lijuan Wang et al. [52]	NA	2015.4	2/F	Bronchopneumonia	2	NA	i.v.gtt.	①	NA	No	No	20min	Anaphylaxis
Lijuan Wang et al. [52]	NA	2015.5	1.5/M	Upper respiratory tract infections	NA	NA	i.v.gtt.	NA	NA	No	No	5min	Anaphylaxis
Xing Du et al. [53]	20050721	NA	31/F	Upper respiratory tract infections	4	NA	i.v.gtt.	①	Azithromycin	No	NA	35min	Anaphylaxis
Xing Du et al. [53]	20050721	NA	42/M	Acute bronchitis	4	NA	i.v.gtt.	①	Penicillin	No	NA	3min	Anaphylaxis
Xing Du et al. [53]	20050721	NA	4.5/F	Upper respiratory tract infections	2	NA	i.v.gtt.	①	Azithromycin	No	NA	3min	Anaphylaxis
Tian Gao et al. [54]	NA	NA	55/F	Severe pneumonia	10	30	i.v.gtt.	④	Cefpirome	No	No	70min	Abdominal distension
Tian Gao et al. [54]	NA	NA	16/M	Psoriasis	8	30	i.v.gtt.	①	10% calcium gluconate injection, VC	No	No	100mL remained	Dizziness of unknown origin
Zhongli Zhang et al. [55]	NA	NA	4/F	Emesis and hypogastralgia	4	NA	i.v.gtt.	②	NA	No	NA	30s	Anaphylaxis
Zhongli Zhang et al. [55]	NA	NA	2/F	Fever of unknown origin	4	NA	i.v.gtt.	①	NA	No	NA	1min	Anaphylaxis
Wei Zhu et al. [56]	20121028	2013.3.25	40/M	Upper respiratory tract infections	8	NA	i.v.gtt.	②	NA	No	No	150mL infused	Anaphylaxis
Wei Zhu et al. [56]	20130202	2013.5.25	10/F	Bronchial asthma	2	NA	i.v.gtt.	①	NA	No	Shrimp	60mL infused	Anaphylaxis
Renze Yang et al. [57]	070118	NA	6/M	Upper respiratory tract infections	2	NA	i.v.gtt.	①	Azithromycin	No	NA	4min	Anaphylaxis
Renze Yang et al. [57]	070118	NA	5/M	Upper respiratory tract infections	2	NA	i.v.gtt.	①	Ceftriaxone sodium	No	NA	40mL infused	Anaphylaxis
Renze Yang et al. [57]	070118	NA	9/M	Amygdalitis	2	NA	i.v.gtt.	①	Cefuroxime axetil	No	NA	10min	Anaphylaxis
Renze Yang et al. [57]	070118	NA	8/F	Bronchitis	2	NA	i.v.gtt.	①	Roxithromycin	No	NA	3min	Anaphylaxis

①5% glucose solution (5% GS); ②normal saline (N.S.); ③10% glucose solution (10% GS); ④5% glucose and sodium chloride injection.

NA: not available; i.v.gtt.: injectio venosa gutta; i.m.: intramuscular; VC: vitamin C injection.

**Table 3:** The primary disease of taking XYP of ADR case reports.

Primary disease	Number of patients	Percentage
Upper respiratory tract infections	17	36.2%
Acute/chronic bronchitis	8	17.0%
Bronchopneumonia	2	4.3%
Amygdalitis	5	10.6%
Pneumonia	4	8.5%
Bronchial asthma	1	2.1%
Bronchiectasia with infections	1	2.1%
Mycoplasma pneumonia	1	2.1%
Acute/chronic bacillary dysentery	1	2.1%
Diarrhea	1	2.1%
Emesis and hypogastralgia	1	2.1%
Adenomesenteritis	1	2.1%
Scald with infections	1	2.1%
Psoriasis	1	2.1%
Fever of unknown origin	2	4.3%
Total	47	100%

**Table 4:** The allergic history of taking XYP of ADR case reports.

Allergic history	Number of patients	Percentage
Not available	26	55.3%
No allergic history	14	29.8%
Cephalosporin	3	6.4%
Penicillins	1	2.1%
Sulfonamides	1	2.1%
Yanhuning injection^ †^	1	2.1%
Shrimp	1	2.1%
Total	47	100%

† Yanhuning injection and XYP injection are both andrographis preparations.

**Table 5:** The drug combination of taking XYP of ADR case reports.

Drug combination		Number/percentage
Antibiotics	Azithromycin	4(21.1%)
	Penicillin	2(10.5%)
	Cefuroxime	2(10.5%)
	Cefoperazone	1(5.3%)
	Ceftriaxone sodium	1(5.3%)
	Ceftezole	1(5.3%)
	Roxithromycin	1(5.3%)
	Mezlocillin sodium	1(5.3%)
	Cefotaxime	1(5.3%)
Antiviral drug	Ribavirin Injection	1(5.3%)
Nutrient	Energy mixture	1(5.3%)
	Vitamin C Injection	1(5.3%)
Antiallergic drug	10% calcium gluconate injection	1(5.3%)
Other traditional Chinese medicine injection (TCMI)	Xingnaojing injection	1(5.3%)
Total		19(100%)

**Table 6:** Details of the symptoms of ADR cases.

Systems	Symptoms (occurrence number and percentage)
Skin structure	Cyanosis of lips (15, 6.7%); cyanosis (10, 4.4%); itch (10, 4.4%); rash (9, 4.0%); flush (6, 2.7%); maculopapule (4, 1.8%); urticaria (1, 0.5%); ecchymosis (1, 0.5%)
Systemic symptoms	Cold limbs (13, 5.8%); pallor (11, 4.9%); hyperhidrosis (9, 4.0%); Chills (8, 3.6%); feebleness (3, 1.3%); fever (1, 0.5%)
Digestive system	Nausea (6, 2.7%); emesis (3, 1.3%); abdominal pain (2, 0.9%)
Respiratory system	Dyspnea (13, 5.8%); short of breath (10, 4.4%); cough (4, 1.8%); polypnea (4, 1.8%); throat itching (1, 0.5%); nasal congestion (1, 0.5%)
Cardiovascular system	Chest congestion (17, 7.6%); drop of blood pressure (15, 6.7%); tachycardia (12, 5.3%); palpitation (5, 2.2%); bradycardia (3, 1.3%)
Nervous system	Irritability (7, 3.1%); coma (4, 1.8%); tremor (4, 1.8%); confusion of consciousness (3, 1.3%); dizziness (3, 1.3%); numbness (2, 0.9%)
Urinary system	Hydrocele (1, 0.5%); oliguria (1, 0.5%)
Application site	Local swelling (2, 0.9%); headache (1, 0.5%)
